# Association between exposure to social media and knowledge of sexual and reproductive health among adolescent girls: evidence from the UDAYA survey in Bihar and Uttar Pradesh, India

**DOI:** 10.1186/s12978-022-01487-7

**Published:** 2022-08-17

**Authors:** Ria Saha, Pintu Paul, Sanni Yaya, Aduragbemi Banke-Thomas

**Affiliations:** 1 Medway Council National Health Service, Kent, UK; 2 Centre for the Study of Regional Development, School of Social Sciences, Jawaharlal Nehru University, New Delhi, India; 3 School of International Development and Global Studies, Faculty of Social Sciences, University of Ottawa, Ottawa, Canada; 4 The George Institute for Global Health, Imperial College London, London, UK; 5 Centre for Vulnerable Children and Families, Institute for Lifecourse Development, University of Greenwich, London, UK

**Keywords:** Adolescents, Adolescent health, Sexual and reproductive health, Social media, Bihar, Uttar Pradesh, India

## Abstract

**Background:**

Poor sexual and reproductive health (SRH) outcomes amongst adolescent girls in India have been associated with inadequate knowledge of SRH. Evidence suggests that social media can promote health-seeking behaviors. Our objective in this study was to determine the association between exposure to social media and SRH knowledge among adolescent girls in Bihar and Uttar Pradesh, India.

**Methods:**

A cross-sectional study was conducted with 10,425 adolescent girls from the UDAYA survey (wave-2, 2018–19). Girls’ exposure to social media was the key predictor, and SRH knowledge of sexual intercourse and pregnancy, contraceptive methods, and HIV/AIDS were outcomes of interest. Multivariable logistic regression models were performed to assess the association between exposure to social media and knowledge of SRH among adolescent girls.

**Results:**

Of the study participants (n = 10,425), 28.0% (n = 3,160) had exposure to social media. Overall, 8.7%, 11.4%, and 6.6% of respondents had sufficient knowledge of sexual intercourse and pregnancy, contraceptive methods, and HIV/AIDS, respectively. Exposure to social media was associated with increased odds of knowledge of sexual intercourse and pregnancy (Odds ratio [OR]: 1.38; 95% confidence interval [CI]: 1.18, 1.61), contraceptive methods (OR: 1.46; 95% CI: 1.27, 1.67), and HIV/AIDS (OR: 2.18; 95% CI: 1.84, 2.58).

**Conclusions:**

Our study shows the potency of exposure to social media in influencing SRH knowledge, which exclusively benefits female adolescents who are educated, residing in urban areas, and from wealthier families. Digital media-focused interventions inclusive of socio-cultural contexts (e.g., strategic investment in education and creating economic opportunities) are crucial to optimize social media's impact on SRH knowledge enhancements.

## Plain language summary

A substantial body of research shows that adolescent girls in India lack adequate sexual and reproductive health (SRH) knowledge. Evidently, this puts them at several health risks associated with early pregnancies, preventable gynecological morbidities such as irregular menstrual patterns, and urethral discharge, among several others. Perpetuated social stigma and lack of agency impede adolescents especially from marginalized communities from accessing accurate, sufficient, and timely SRH information from their immediate household members and healthcare providers at local service points. This adversely affects their health-seeking behaviors and ultimately results in avertable poor reproductive health outcomes. A growing body of literature highlights social media platforms (i.e., Facebook, Instagram, Twitter, etc.) as preferred modalities to gain SRH information among adolescents. Against this backdrop, we assessed the intricate association between social media exposure and SRH knowledge (across three dimensions: sexual intercourse and pregnancy, contraceptive methods, and HIV/AIDS) among adolescent girls in Bihar and Uttar Pradesh, India. We tested for association between several socio-demographic factors and SRH knowledge. Findings indicate that adolescents who were exposed to social media platforms were likely to have more SRH knowledge compared to those who were not. Socio-demographic factors like place of residence, for example residing in urban areas, higher levels of education and high wealth index acted as facilitators of social media exposure as well as SRH knowledge.

## Background

Adolescence is a critical transition period from childhood to adulthood that determines the health and wellbeing of an individual in the latter part of life. India is the country with the largest number of adolescents aged 10 to 19 years globally (253 million), of which nearly 50% are females [1]. Recognizing the importance of protecting the sexual and reproductive health (SRH) and wellbeing of adolescents, India has implemented strategies including the Adolescent Reproductive and Sexual Health Strategy (ARSH) (2005–2013) and *Rashtriya Kishor Swasthya Karyakram,* or National Adolescent Health Programme (2014–present, replacing ARSH). Both were designed to support and escalate the demand for essential adolescent health services [2, 3]. Despite these initiatives, many adolescent girls face structural barriers to accessing accurate and timely information on their SRH, resulting in around 2.4 million adolescents with an unmet need for modern contraception for instance [4–7].

A cross-sectional survey conducted in rural Jharkhand, India, indicated a complete lack of essential SRH knowledge, where 94% of young women claimed to have never received any information regarding critical sexual health-related matters such as abortion and contraception [8]. Another study from Jharkhand highlighted that only 30% of participants were cognizant of contraception, 24% expressed sufficient knowledge about oral contraception, and even fewer, e.g., about 10% of married girls had ever used contraceptive methods to delay early childbearing [9].

The absence of or insufficient SRH information negatively affects fertility decisions and predisposes young women to adverse pregnancy outcomes such as maternal/neonatal mortality, low birth weight, and premature births. It also increases vulnerability to adverse effects following use of medications. For example, having ingested wrong doses of medicines. It also predisposes young women to preventable gynecological morbidities such as irregular menstrual patterns, urethral discharge, and burning urination, which result in poor health outcomes [10–15]. As per available evidence, social stigma, Lack of female autonomy arising from limited individual financial resources and restricted social mobility prevent girls specifically from disadvantaged communities to access accurate, sufficient, and timely SRH information from healthcare providers at local service points [6, 12, 16–18]. Restrictive social conservative norms prevailing in rural India result in insufficient and inadequate parent-adolescent communication which majorly restricts girls from gaining and developing SRH knowledge from parents and immediate household members.

An emerging body of literature suggests a paradigm shift in the information-seeking behavior of adolescents where they are likely to receive SRH information from traditional mass media such as print, television, and radio to social media platforms such as Facebook and WhatsApp [8, 14, 19–21]. Considering there have been efforts to expand mobile health (mHealth) centric health communication in India through direct text messages and phone calls, questions remain about the capacity and strength of social media platforms in shaping SRH knowledge among adolescent girls [22–24]. Given the high presence of social media users in India [22], it is vital to assess its potential reach in propagating accurate SRH-related information. Against this backdrop, we aim to assess the association between social media exposure and SRH knowledge among adolescent girls in Bihar and Uttar Pradesh, India. Since adolescent girls consistently face discriminatory service provision and are highly vulnerable to adverse SRH outcomes, it is imperative to understand this association, which could adequately empower girls with sufficient SRH information.

## Methods

### Study setting

This study was based on data collected from two Indian states, namely Bihar and Uttar Pradesh. Uttar Pradesh is the most populous state in the country, with a population of 200 million, accounting for 17% of India’s population. With a population of 104 million, Bihar is the third-largest state in terms of population, accounting for 9% of India’s population [25]. In both states, most of the population live in rural areas, with just 11% and 22% of the people residing in urban areas of Bihar and Uttar Pradesh, respectively, in 2011 [25]. Bihar and Uttar Pradesh are the poorest among all Indian states, with 34% of the population in Bihar and 29% in Uttar Pradesh living below the poverty line in 2011–12 [26]. Female literacy in both states is below the nation’s average (65%), where 52% of females in Bihar and 57% of females in Uttar Pradesh are literate [25]. Child marriages and adolescent pregnancies are widespread in both states, with 41% of women in Bihar and 16% of women aged 20–24 years in Uttar Pradesh being married before 18 years [27]. About 11% and 3% of pregnancies occur during adolescence in Bihar and Uttar Pradesh, respectively [27]. In terms of female ownership of mobile phones and access to internet, these states are one of the least performers among Indian states. In Bihar, about one-half of women (51%) own a mobile phone and 21% of them accessed the internet. In Uttar Pradesh, fewer than half of women (47%) had a mobile phone and 59% had accessed the internet in 2019–21 [27]. Evidence also suggests that the use of SRH services is substantially low in these states [28]. Fewer than half of the women of reproductive age (44–45%) used any modern methods of contraception to avoid or delay pregnancy in both states during the period 2019–2021 [27].

### Data source

Data for this cross-sectional study was drawn from the “Understanding the Lives of Adolescents and Young Adults (UDAYA)” survey conducted by the Population Council. This data is a unique longitudinal survey of adolescent boys and girls (aged 10–19 years) in Bihar and Uttar Pradesh. The UDAYA project provides robust insights into factors that determine the transition from adolescence to early adulthood. The survey includes information about the lives of adolescents and young adults such as education, economic activity, knowledge, and awareness about SRH, mass media and social media exposure, aspirations, agency, gender role attitudes, romantic and sexual relationships, transition to marriage and parenthood, health, and health-seeking behavior [28].

The UDAYA survey adopted a multi-stage systematic sampling design to draw the sample from rural and urban areas. A total of 150 primary sampling units (PSUs)—villages in rural areas and census wards in urban areas—were selected. The 2011 census list of villages and wards served as the sampling frame. Complete mapping and household listing operation were carried out in each selected PSU. Based on this list, a PSU was divided into two nearly equal segments—one segment was randomly chosen for conducting interviews with females and the other for interviews with males. In each PSU, households were selected with equal probability from the list using systematic sampling. Only one respondent from each category was interviewed within each household, resulting in a maximum of three interviews per household—one younger girl, one unmarried older girl, and one married older girl in the female segment, and one younger boy, one older boy, and one married older girl in the male segment. If a household had more than one respondent from a single category, the Kish table was used to select one respondent randomly, and no replacement of the respondent was allowed [28, 29].

In the end, 20,594 interviews (5,969 boys and 14,625 girls) were completed in both states in 2015–16 (wave-1). Among these 20,994 eligible respondents, UDAYA again re-interviewed 4,567 boys and 12,251 girls in 2018–19. The reasons for the loss to follow-up were migration of the participant, refusal to be re-interviewed by the parent or guardian, researchers’ inability to track the household, and refusal by the participant. Participants who gave an inconsistent response to age and education were excluded (3%) from the sample. Thus, the final sample in the follow-up survey (wave-2) comprised 4,428 boys and 11,864 girls, with an effective follow-up rate of 74% for boys and 81% for girls.

### Ethical considerations

This study uses publicly available secondary data and as such did not any ethical approval from an institutional review board. The UDAYA survey which informed this study was approved by the ethical review board of Population Council, New Delhi. Informed consent was obtained from all subjects at the time of the survey. Informed consent was sought from a parent or guardian of unmarried adolescents aged 10–17 years [28, 29].

### Process of sub-sample selection for this study

**Fig. 1:**
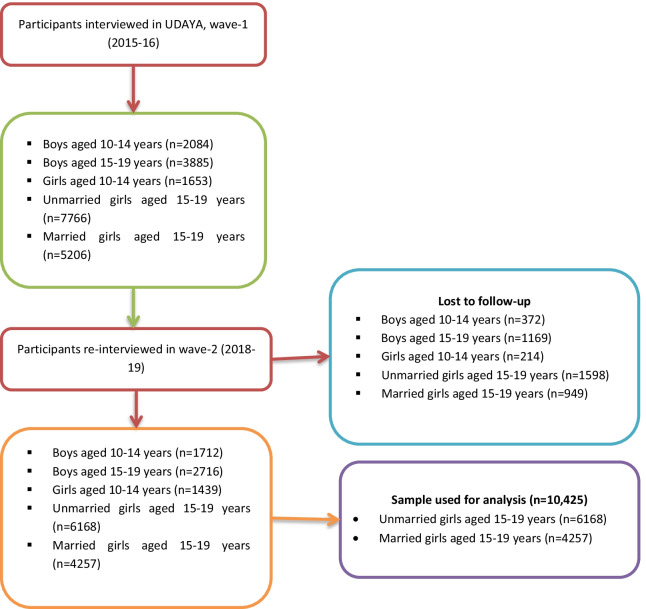
Flow chart showing selection of study participants

In wave-2 (2018–19), 16,292 adolescent boys and girls were successfully re-interviewed. Since questions on access to social media and knowledge of SRH were asked among respondents aged 15 years or older, we limited our sample to older cohorts of girls. Therefore, we excluded the sample of 4428 unmarried boys (aged 10–14 years and 15–19 years) and 1439 unmarried younger girls (aged 10–14 years). For this study, we drew data from a subset of 10,425 samples from wave-2 (2018–19), based on the cohorts of unmarried and married girls (aged 15–19 years at wave-1, 2015–16) who were 18–22 years at wave-2, 2018–19. Figure 1 illustrates the process of selecting the sub-sample for conducting this research.

### Measures

#### Knowledge of SRH

Knowledge of SRH was the outcome of interest in this study. We included three outcome variables in this study: (1) knowledge about sexual intercourse and pregnancy, (2) knowledge of contraceptive methods, and (3) knowledge of HIV/AIDS. We categorized each outcome variable into girls with sufficient knowledge (coded as ‘1’) and girls with insufficient knowledge (coded as ‘0’).

Knowledge about sexual intercourse and pregnancy was assessed from two specific questions: (1) can a woman get pregnant the first time she has sexual intercourse? And (2) are there certain days when a woman is more likely to become pregnant if she has sexual relations from one menstrual period to the next? If respondents answered ‘yes’, they were further asked about the timing of pregnancy: is this time just before her period begins, during her period, right after her period has ended, or halfway between two periods? Participants who responded ‘halfway between two periods’ were considered to have correct knowledge about the timing of pregnancy. A dichotomous variable was constructed where participants who had the correct knowledge on both questions were considered to have sufficient knowledge (coded as ‘1’), otherwise having insufficient knowledge (coded as ‘0’).

Knowledge of contraceptive methods was assessed from responses provided to the three questions on family planning methods to avoid or delay pregnancy. As per the survey tool, participants were initially asked whether they had heard of contraceptive methods. Participants who responded ‘yes’ were further asked the three following questions: (1) How often should a woman take oral pills to prevent pregnancy? (2) How soon after sexual intercourse should these pills be taken? And (3) one condom can be used for how many acts of sexual intercourse? Participants who answered that a woman should take oral pills everyday/weekly, take emergency contraceptive pills within three days/72 h after sexual intercourse, and that one condom could be used for one sexual intercourse were assessed to have provided correct answers to the respective questions on contraceptive methods. Those who had given the right answers to any two questions were considered to have sufficient knowledge (coded as ‘1’), otherwise insufficient knowledge (coded as ‘0’).

Knowledge of HIV/AIDS was assessed with five questions—three common misconceptions about HIV (the AIDS virus being transmitted through mosquito bites, sharing food with a person who has AIDS, and hugging someone who has AIDS) and two major ways of preventing HIV (people reduce their chances of getting the AIDS virus by having just one sex partner and using a condom every time they have sex). Respondents who rejected these misconceptions about the AIDS virus and knew the ways of preventing HIV were assessed to have provided the correct answers to these questions about HIV/AIDS. Those who had the correct answers to all five questions were considered to have sufficient knowledge (coded as ‘1’), otherwise insufficient knowledge (coded as ‘0’).

#### Exposure to social media

Exposure to social media among females was considered the key predictor variable to assess its association with the knowledge of SRH. In UDAYA (wave 2, 2018–19), respondents were asked about their use of social media (e.g., Facebook, Twitter, WhatsApp, WeChat, Instagram, Jio chat, Messenger chat, and any other) in the last three years, based on data collected in the UDAYA survey. The information was collected as multiple responses from the participants. Based on these responses, we constructed a binary variable where participants who used any social media platforms were considered to have exposure to social media (coded ‘1’). In contrast, those who do not have internet access, do not know about social media, and have not used social media in the last three years were not exposed to social media (coded as ‘0’).

#### Explanatory variables

We included nine socio-demographic characteristics in the analysis that potentially influence girls’ exposure to social media and their knowledge of SRH. The selection of these explanatory variables is based on previous studies [8, 43, 44]. These variables include the age of respondents (15–19 years), place of residence (urban and rural), caste (Scheduled Caste [SC], Scheduled Tribe [ST], Other Backward Class [OBC], and non-SC/ST/OBC [General caste]), religion (Hindu, Muslim, others), educational attainment (no education, primary, secondary, higher), paid work in last 12 months (no and yes), marital status (unmarried and married), wealth quintile (poorest, poorer, middle, richer, and richest), and states (Uttar Pradesh and Bihar). Table 1 provides a brief description of the variables included in this study.

**Table 1:** Description of variables included in the study

Variable name	Variable type	Coding/description
*Outcome variables*		
Knowledge of sexual intercourse & pregnancy	Binary	Insufficient knowledge = 0; Sufficient knowledge = 1
Knowledge of contraceptive methods	Binary	Insufficient knowledge = 0; Sufficient knowledge = 1
Knowledge of HIV/AIDS	Binary	Insufficient knowledge = 0; Sufficient knowledge = 1
*Main predictor variable*		
Exposure to social media	Binary	Not exposed = 0; Exposed = 1
*Explanatory variables*		
Age of respondents	Categorical	15–19 in wave-1 (2015–16) (they were 18–22 in wave-2, 2018–19)
Place of residence	Categorical	Urban = 1; Rural = 2
Caste	Categorical	SC = 1; ST = 2; OBC = 3; General = 4
Religion	Categorical	Hindu = 1; Muslim = 2; Others = 3
Education	Categorical	Completed years of schooling categorized into four groups: No education/illiterate = 1; Primary (0–5 years of schooling) = 2; Secondary (6–12 years of schooling) = 3; Higher (13 + years of schooling) = 4
Paid work in last year	Binary	No = 0; Yes = 1
Marital status	Categorical	Unmarried = 1 Married = 2
Wealth quintile	Categorical	Household wealth is measured from possession of durable goods and amenities such as agricultural land, access to toilet and electricity, television, car, bicycle, sewing machine, etc. Wealth scores—ranging from 3 to 55—divided into 5 quintiles, from the lowest (poorest) to highest (richest)
States	Categorical	Uttar Pradesh = 1; Bihar = 2

### Analysis

We performed descriptive statistics to show the distribution of study participants by socio-demographic characteristics. The percentage distribution was estimated to observe the differentials in exposure to social media and the knowledge of SRH (sufficient knowledge vs insufficient knowledge) by the selected explanatory variables. The appropriate sample weight was used to estimate the percentages, and differences were tested using Pearson’s Chi-square statistic.

A multivariable binary logistic regression model was applied to examine socio-demographic determinants of exposure to social media. Multivariable logistic regression models were performed for each outcome variable to assess the association between exposure to social media and SRH. The results of regression models were presented by odds ratio (OR) with a 95% confidence interval (CI). P-values indicated the level of significance. All statistical analyses were executed using STATA SE version 16.0 (StataCorp, College Station, TX, USA).

## Results

### Characteristics of respondents

A total of 10,425 adolescent girls were analyzed in the present study, with a median age of 17 years (SD =  ± 1.4). The majority lived in rural areas (84.3%) and were Hindu (79.4%). Over half of the participants (57.6%) belonged to the OBC category. Sixty-two per cent of the girls were at the secondary level of education, and nearly one-quarter of them (23.2%) were involved in any paid work during the last year. Over half of the respondents (54%) were unmarried at data collection. More than two-fifths of the participants (43.3%) belonged to the bottom 40% of the household wealth quintile. The representation of the study sample was almost similar in both states (53% in Bihar vs 47% in Uttar Pradesh (Table 2).

**Table 2:** Sample distribution of study participants, UDAYA (wave-2, 2018–19)

Variables	Exposure to social media				Total (N = 10,425)	
	Not exposed (Total = 7265)		Exposed (Total = 3160)			
	Un-weighted n	Weighted %	Un-weighted n	Weighted %	Un-weighted n	Weighted %
*Age of respondents*						
15	1196	23.5	569	20.7	1765	22.7
16	1273	21.8	595	22.4	1868	22.0
17	1296	18.5	641	21.1	1937	19.2
18	1765	19.6	721	19.8	2486	19.6
19	1735	16.6	634	16.0	2369	16.5
*Place of residence*						
Urban	2464	11.1	1831	27.3	4295	15.7
Rural	4801	88.9	1329	72.7	6130	84.3
*Caste*						
SC	1966	26.1	472	15.2	2438	23.1
ST	63	1.0	17	0.8	80	0.9
OBC	4236	58.8	1772	54.6	6008	57.6
General	936	14.2	843	29.4	1779	18.4
*Religion*						
Hindu	5696	79.3	2403	79.6	8099	79.4
Muslim	1510	19.9	687	18.3	2197	19.4
Others	59	0.9	70	2.2	129	1.2
*Education*						
Illiterate	1434	14.5	99	2.0	1533	11.0
Primary	935	11.7	111	2.8	1046	9.2
Secondary	4179	62.1	1826	60.4	6005	61.6
Higher	717	11.7	1124	34.8	1841	18.2
*Paid work in last year*						
No	5734	77.3	2437	75.6	8171	76.8
Yes	1531	22.7	723	24.4	2254	23.2
*Marital status*						
Unmarried	2801	51.1	1816	61.5	4617	54.0
Married	4464	48.9	1344	38.5	5808	46.0
*Wealth quintile*						
Poorest	2272	29.8	244	8.4	2516	23.8
Poorer	1572	22.3	362	12.2	1934	19.5
Middle	1671	21.4	677	21.5	2348	21.4
Richer	1041	14.5	796	24.2	1837	17.2
Richest	709	11.9	1081	33.8	1790	18.0
*States*						
Uttar Pradesh	3412	47.9	1343	44.7	4755	47.0
Bihar	3853	52.1	1817	55.3	5670	53.0

Percentages are weighted using combining weight for married and unmarried girls aged 15–19 years at wave-1 for both the states

### Knowledge of SRH

**Fig. 2:**
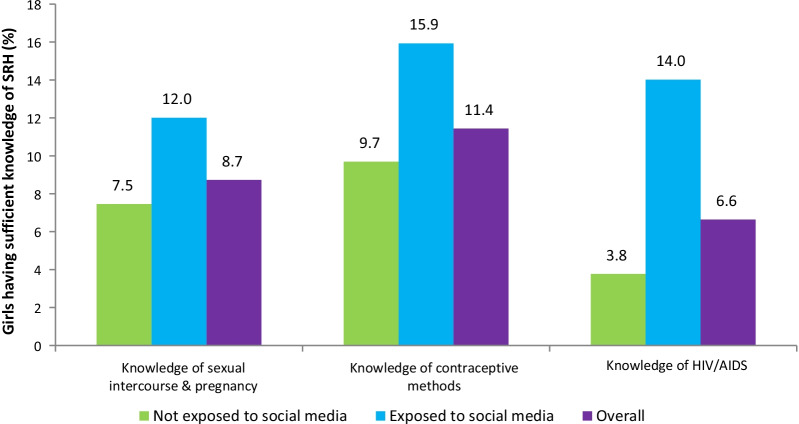
Percentage distribution of sufficient knowledge of SRH (%) by exposure to social media, UDAYA (wave-2, 2018–19)

Overall, 8.7%, 11.4%, and 6.6% of adolescent girls had sufficient knowledge of sexual intercourse and pregnancy, contraceptive methods, and HIV/AIDS, respectively. Furthermore, a significantly higher proportion of girls exposed to social media had sufficient knowledge across all three indices of SRH than those who were not (Fig. 2).

### Exposure to and use of social media

**Fig. 3:**
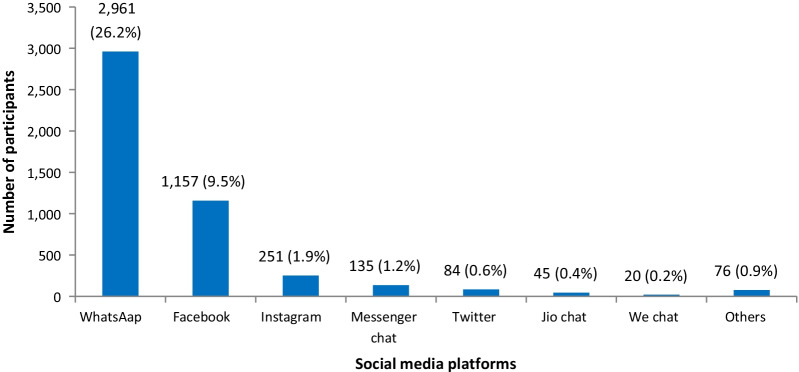
Use of social media platforms by the study participants (n = 3160, 28.0%), UDAYA (wave-2, 2018–19)

Of the total study participants, 28% (n = 3160) had exposure to social media. Among various social media platforms, WhatsApp (26.2%) was the most used platform, followed by Facebook (9.5%) and Instagram (1.9%) (Fig. 3).

### Differentials and determinants of exposure to social media

Participants residing in urban areas were twice as exposed as those in rural areas (48.8% vs 24.1%, *p* < 0.001). Exposure to social media was higher among girls who belonged to the general caste (44.5%) than those from SC (18.4%), ST (24%), and OBC (26.4%) (*p* < 0.001). Participants’ exposure to social media significantly increased with their increasing levels of education (*p* < 0.001). Respondents involved in paid work in the last year had a 2%-point greater exposure to social media than those not involved (*p* = 0.04). The proportionate share of social media exposure was higher among unmarried girls than married ones (31.9% vs 23.5%, *p* < 0.001). Exposure to social media substantially differed across wealth quintiles (*p* < 0.001), where only one in ten respondents from the poorest quintile was exposed to social media (9.9%). In contrast, more than half of the richest quintile had exposure to social media (52.4%). Girls from Bihar had higher exposure to social media than those from Uttar Pradesh (29.2% vs 26.6%, *p* < 0.001) (Table 3).

**Table 3:** Participants’ exposure to social media by socio-demographic characteristics, UDAYA (wave-2, 2018–19)

Variables	Exposure to social media		
	Not exposed (%)	Exposed (%)	*p*-value
*Age of respondents*			< 0.001
15	74.5	25.6	
16	71.5	28.5	
17	69.2	30.8	
18	71.8	28.2	
19	72.7	27.3	
*Place of residence*			< 0.001
Urban	51.2	48.8	
Rural	75.9	24.1	
*Caste*			< 0.001
SC	81.6	18.4	
ST	76.0	24.0	
OBC	73.6	26.4	
General	55.6	44.5	
*Religion*			< 0.001
Hindu	71.9	28.1	
Muslim	73.6	26.4	
Others	50.6	49.4	
*Education*			< 0.001
Illiterate	94.9	5.2	
Primary	91.4	8.6	
Secondary	72.6	27.5	
Higher	46.3	53.7	
*Paid work in last year*			0.04
No	72.4	27.6	
Yes	70.6	29.5	
*Marital status*			< 0.001
Unmarried	68.1	31.9	
Married	76.5	23.5	
*Wealth quintile*			< 0.001
Poorest	90.1	9.9	
Poorer	82.5	17.5	
Middle	72.0	28.0	
Richer	60.7	39.3	
Richest	47.6	52.4	
*States*			< 0.001
Uttar Pradesh	73.4	26.6	
Bihar	70.8	29.2	

*P*-values are derived from Pearson’s Chi-square statistic assessing the test of association for the differences in proportions between the exposed and non-exposed groups

Amongst all the included socio-demographic variables, education and household wealth had the strongest associations with social media exposure. Girls with higher educational attainment were 12 times (OR: 12.01; 95% CI: 9.36, 15.41) more likely to be exposed to social media than illiterate ones. Respondents from the richest wealth quintile were associated with six times (OR: 6.13; 95% CI: 5.11, 7.37) higher odds of social media exposure compared to the poorest quintile. The odds of exposure to social media were 50% lower among rural girls (OR: 0.50; 95% CI: 0.46, 0.56) compared to those living in urban areas. The odds of exposure to social media were higher among girls from the general caste (OR: 1.74; 95% CI: 1.48, 2.05) as compared to SC girls. Adolescents involved in paid work were more likely to be exposed to social media (OR: 1.23; 95% CI: 1.08, 1.39) than those not involved. Girls from Bihar were twice more likely to be exposed to social media (OR: 2.02; 95% CI: 1.82, 2.25) than those from Uttar Pradesh (Table 4).

**Table 4:** Multivariable binary logistic regression models assessing the odds of social media exposure among adolescent girls aged 15–19 years, UDAYA (wave-2, 2018–19)

Variables	OR	*P*-value	95% CI	
			LB	UB
*Age of respondents*				
15 (ref.)				
16	0.95	0.511	0.81	1.11
17	0.91	0.287	0.78	1.08
18	0.81	0.015	0.69	0.96
19	0.74	0.001	0.62	0.88
*Place of residence*				
Urban (ref.)				
Rural	0.50	< 0.001	0.46	0.56
*Caste*				
SC (ref.)				
ST	0.69	0.233	0.38	1.27
OBC	1.17	0.020	1.03	1.34
General	1.74	< 0.001	1.48	2.05
*Religion*				
Hindu (ref.)				
Muslim	1.10	0.138	0.97	1.25
Others	1.69	0.229	0.72	3.96
*Education*				
Illiterate				
Primary	1.68	0.001	1.25	2.25
Secondary	4.42	< 0.001	3.54	5.53
Higher	12.01	< 0.001	9.36	15.41
*Paid work in last year*				
No (ref.)				
Yes	1.23	0.001	1.08	1.39
*Marital status*				
Unmarried (ref.)				
Married	1.02	0.760	0.9	1.15
*Wealth quintile*				
Poorest (ref.)				
Poorer	1.56	< 0.001	1.3	1.87
Middle	2.21	< 0.001	1.86	2.62
Richer	3.73	< 0.001	3.13	4.45
Richest	6.13	< 0.001	5.11	7.37
*States*				
Uttar Pradesh (ref.)				
Bihar	2.02	< 0.001	1.82	2.25

*OR* odds ratio, *CI* confidence interval, *LB* lower bound, *UB* upper bound, *ref.* reference category

### Association between exposure to social media and knowledge of SRH

We found a higher share of sufficient knowledge about sexual intercourse and pregnancy (12% vs 7.5%, *p* < 0.001), contraceptive methods (15.9% vs 9.7%, *p* < 0.001), and HIV/AIDS (14% vs 3.8%, *p* < 0.001) among girls who were exposed to social media than those who were not exposed. The sufficient knowledge of SRH under all three indices increased with the age of the participants. Girls living in urban areas had greater knowledge of contraceptive methods (13.7% vs 11%, *p* < 0.001) and HIV/AIDS (13.5% vs 5.4%, *p* < 0.001) than their rural counterparts. Adolescent girls who belonged to the general caste were found to be significantly more knowledgeable about sexual intercourse and pregnancy (9.3%) and HIV/AIDS (10.4%) compared to the girls from disadvantaged castes (*p* = 0.003). For contraceptive methods, there was a higher proportion of ST girls who had more knowledge (19.8%) compared to the girls from other caste groups (*p* < 0.001). Girls having higher education were more knowledgeable about sexual intercourse and pregnancy (9.3% vs 6.3%, *p* < 0.001), contraceptive methods (17% vs 9.1%, *p* < 0.001), and HIV/AIDS (17.7% vs 0.7%, *p* < 0.001) when compared with illiterate girls. When compared to unmarried girls, married girls reported a significantly higher percentage of sufficient knowledge of sexual intercourse and pregnancy (13.2% vs 4.9%, *p* < 0.001) and contraceptive methods (16% vs 7.6%, *p* < 0.001); however, unmarried girls were more informed about HIV/AIDS (7.6% vs 5.5%, *p* < 0.001). Compared to girls from the poorest quintile, a considerably higher percentage of girls from the richest quintile had sufficient knowledge of sexual intercourse and pregnancy (12.3% vs 6%, *p* < 0.001), contraceptive methods (16.1% vs 8.2%, *p* < 0.001), and HIV/AIDS (15.1% vs 1.9%, *p* < 0.001). Girls residing in Bihar had a greater proportion of sufficient knowledge about sexual intercourse and pregnancy (10.7% vs 6.5%, *p* < 0.001) and contraceptive methods (12.2% vs 10.6%, *p* < 0.001) than those residing in Uttar Pradesh (Table 5).

**Table 5:** Percentage distribution of knowledge of sexual intercourse and pregnancy, contraceptive methods and HIV/AIDS by the key predictor and explanatory variables among adolescent girls aged 15–19 years, UDAYA (wave-2, 2018–19)

Variables	Knowledge of sexual intercourse and pregnancy			Knowledge of contraceptive methods			Knowledge of HIV/AIDS		
	Insufficient	Sufficient	*p*-value	Insufficient	Sufficient	*p*-value	Insufficient	Sufficient	*p*-value
*Exposure to social media*			< 0.001			< 0.001			< 0.001
Not exposed	92.5	7.5		90.3	9.7		96.2	3.8	
Exposed	88.0	12.0		84.1	15.9		86.0	14.0	
*Age of respondents*			< 0.001			< 0.001			< 0.001
15	93.6	6.4		92.6	7.4		95.4	4.6	
16	93.1	6.9		92.9	7.1		94.6	5.4	
17	91.1	8.9		88.5	11.5		93.5	6.5	
18	89.4	10.6		85.7	14.3		91.4	8.6	
19	88.0	12.0		80.6	19.4		91.2	8.8	
*Place of residence*			0.430			< 0.001			< 0.001
Urban	91.2	8.8		86.3	13.7		86.5	13.5	
Rural	91.3	8.7		89.0	11.0		94.6	5.4	
*Caste*			0.003			< 0.001			< 0.001
SC	92.2	7.8		91.0	9.0		96.2	3.8	
ST	93.4	6.6		80.2	19.8		97.3	2.7	
OBC	91.1	8.9		88.5	11.5		93.5	6.5	
General	90.7	9.3		86.3	13.7		89.6	10.4	
*Religion*			0.213			0.088			< 0.001
Hindu	91.1	8.9		88.3	11.7		93.4	6.6	
Muslim	92.2	7.8		90.0	10.1		93.6	6.5	
Others	88.7	11.3		84.1	15.9		85.1	14.9	
*Education*			0.001			< 0.001			< 0.001
Illiterate	93.7	6.3		90.9	9.1		99.3	0.7	
Primary	92.7	7.3		92.8	7.2		98.2	1.8	
Secondary	90.8	9.2		89.2	10.8		94.8	5.2	
Higher	90.7	9.3		83.0	17.0		82.4	17.7	
*Paid work in last year*			0.001			0.057			0.019
No	90.7	9.3		88.3	11.7		93.7	6.3	
Yes	93.1	6.9		89.6	10.4		92.3	7.7	
*Marital status*			< 0.001			< 0.001			< 0.001
Unmarried	95.1	4.9		92.4	7.6		92.4	7.6	
Married	86.8	13.2		84.0	16.0		94.5	5.5	
*Wealth quintile*			< 0.001			< 0.001			< 0.001
Poorest	94.0	6.0		91.8	8.2		98.1	1.9	
Poorer	91.6	8.4		89.9	10.1		96.2	3.8	
Middle	92.2	7.8		88.7	11.3		94.6	5.4	
Richer	89.8	10.2		87.3	12.7		90.8	9.2	
Richest	87.7	12.3		84.0	16.1		84.9	15.1	
*States*			< 0.001			< 0.001			0.389
Uttar Pradesh	93.5	6.5		89.4	10.6		92.6	7.4	
Bihar	89.3	10.7		87.8	12.2		94.1	5.9	

Multivariable logistic regression analyses showed that exposure to social media was significantly associated with higher odds of knowledge of sexual intercourse and pregnancy (OR: 1.38; 95% CI: 1.18, 1.61), contraceptive methods (OR: 1.46; 95% CI: 1.27, 1.67), and HIV/AIDS (OR: 2.18; 95% CI: 1.84, 2.58). Among socio-demographic characteristics, educational attainment imparted a strong positive association with all three indices of SRH. Specifically, girls with higher education had almost nine-fold greater odds of knowledge about HIV/AIDS (OR: 8.73; 95% CI: 5.18, 14.72) than uneducated ones. Adolescent girls living in rural areas were less likely to have knowledge of contraceptive methods (OR: 0.82; 95% CI: 0.72, 0.93) and HIV/AIDS (OR: 0.63; 95% CI: 0.53, 0.74) than those from urban areas. Married girls were more likely to have correct information about sexual intercourse and pregnancy (OR: 3.34; 95% CI: 2.77, 4.02) and contraceptive methods (OR: 2.85; 95% CI: 2.44, 3.34) than unmarried ones. Household wealth also had a significant positive association with all three indices of SRH, where girls from the richest quintile were 1.75 (95% CI: 1.37, 2.23), 2.05 (95% CI: 1.66, 2.54), and 3.07 (95% CI: 2.21, 4.27) times more likely to know about sexual intercourse and pregnancy, contraceptive methods, and HIV/AIDS (Table  6).

**Table 6:** Multivariable logistic regression models assessing the association between girls' exposure to social media and knowledge of sexual intercourse and pregnancy, contraceptive methods and HIV/AIDS, UDAYA (wave-2, 2018–19)

	Model 1 = Knowledge of sexual intercourse and pregnancy				Model 2 = Knowledge of contraceptive methods				Model 3 = Knowledge of HIV/AIDS			
Variables	OR	*p*-value	95% CI		OR	*p*-value	95% CI		OR	*p*-value	95% CI	
			LB	UB			LB	UB			LB	UB
*Exposure to social media*												
Not exposed (ref.)												
Exposed	1.38	< 0.001	1.18	1.61	1.46	< 0.001	1.27	1.67	2.18	< 0.001	1.84	2.58
*Age of respondents*												
15 (ref.)												
16	0.96	0.782	0.74	1.26	0.97	0.804	0.76	1.23	0.93	0.602	0.71	1.22
17	1.10	0.465	0.85	1.42	1.29	0.028	1.03	1.62	1.02	0.896	0.78	1.33
18	1.21	0.135	0.94	1.55	1.37	0.005	1.10	1.71	1.24	0.104	0.96	1.62
19	1.27	0.065	0.99	1.63	1.79	< 0.001	1.44	2.24	1.51	0.003	1.15	1.98
*Place of residence*												
Urban (ref.)												
Rural	1.00	0.968	0.87	1.16	0.82	0.001	0.72	0.93	0.63	< 0.001	0.53	0.74
*Caste*												
SC (ref.)												
ST	0.69	0.442	0.27	1.76	1.91	0.027	1.08	3.38	0.55	0.327	0.16	1.82
OBC	1.15	0.121	0.96	1.38	1.00	0.974	0.86	1.17	1.12	0.321	0.90	1.39
General	1.32	0.020	1.05	1.68	1.21	0.059	0.99	1.48	1.22	0.128	0.95	1.56
*Religion*												
Hindu (ref.)												
Muslim	1.14	0.146	0.96	1.37	1.01	0.876	0.86	1.19	0.97	0.788	0.80	1.18
Others	1.16	0.811	0.34	4.02	0.83	0.737	0.28	2.49	0.96	0.940	0.31	2.97
*Education*												
Illiterate (ref.)												
Primary	1.00	0.985	0.74	1.36	0.99	0.922	0.76	1.28	1.79	0.073	0.95	3.38
Secondary	1.54	< 0.001	1.24	1.93	1.33	0.004	1.10	1.61	4.45	< 0.001	2.70	7.35
Higher	1.70	< 0.001	1.27	2.26	1.91	< 0.001	1.49	2.43	8.73	< 0.001	5.18	14.72
*Paid work in last year*												
No (ref.)												
Yes	1.04	0.672	0.87	1.24	1.13	0.124	0.97	1.31	1.17	0.095	0.97	1.40
*Marital status*												
Unmarried (ref.)												
Married	3.34	< 0.00!	2.77	4.02	2.85	< 0.001	2.44	3.34	1.08	0.440	0.89	1.30
*Wealth quintile*												
Poorest (ref.)												
Poorer	1.24	0.055	1.00	1.55	1.16	0.139	0.95	1.42	1.51	0.022	1.06	2.14
Middle	1.21	0.092	0.97	1.50	1.23	0.037	1.01	1.49	1.71	0.001	1.23	2.37
Richer	1.45	0.002	1.15	1.83	1.61	< 0.001	1.31	1.97	2.54	< 0.001	1.83	3.52
Richest	1.75	< 0.001	1.37	2.23	2.05	< 0.001	1.66	2.54	3.07	< 0.001	2.21	4.27
*States*												
Uttar Pradesh (ref.)												
Bihar	1.34	< 0.001	1.16	1.56	1.17	0.015	1.03	1.33	1.16	0.065	0.99	1.37

*OR* odds ratio, *CI* confidence interval, *LB* lower bound, *UB* upper bound, *ref.* reference category

## Discussion

We find that exposure to social media has a significant positive association with the knowledge of sexual intercourse and pregnancy, contraceptive methods, and HIV/AIDS. This association was retained even after adjusting for socio-demographic factors in multivariate analyses. Amongst all the socio-demographic factors, education and wealth index imparted the strongest association, emphasizing education and household wealth as important facilitators of social media accessibility and SRH knowledge acquisition. In contrast, rural residence emerged as the most prominent deterrent to social media accessibility and SRH knowledge gain. Although marital status showed no association with exposure to social media, married adolescent girls were significantly well informed about sexual intercourse, and pregnancy, and contraceptive methods.

Broadly, our comprehensive analyses provided a nuanced understanding of the association between exposure to social media and SRH knowledge at the intersection of pervasive context-specific/regional structural inequities. Inequities across socio-economic groups substantially hinder disadvantaged adolescents from accessing social networking platforms and deny all possible associated direct and indirect health benefits. For example, girls from rural areas, less educated, and belonging to economically impaired families were significantly less likely to engage in any social media platforms and were found to be poorly informed about SRH. Low social media exposure in rural areas is mainly a potential manifestation of the urban/rural digital divide and pervasive gender inequity, restricting girls from internet-facilitated social media connectivity. Raj et al. highlighted the presence of low digital connectivity in the rural areas of Bihar and Uttar Pradesh (India), demonstrating its association with inequitable gender role beliefs [30].

Regarding education, less-educated adolescent girls are likely to have lower self-efficacy and less agency/autonomy to participate in household decision-making, potentially resulting in reduced access to digital connectivity. Evidence from rural Madhya Pradesh, India, showed higher digital technology engagement and responsiveness to digital health interventions among beneficiaries (pregnant and postpartum women of reproductive age 15–49 years) who were comparatively more educated [31, 32]. Higher education opens doors to economic opportunities. Educated girls are more likely to engage in paid jobs, leading to financial independence. The greater odds of social media exposure among girls involved in paid work imply economic empowerment (personal mobile phone ownership/internet access) related to freedom of movement as possible factors. These findings are in parallel with earlier studies conducted by Raj et al. (2021) and Scott et al. (2021) [30, 31]. Affordability and the capacity to sustainably support the functionality of new-age digital devices supposedly enable wealthier adolescents to get more access to cellular devices and exposure to social media compared to their poorest counterparts [30, 32].

The multivariate regression models conducted in our study showed a strong positive association between exposure to social media and SRH knowledge, highlighting social media as an important medium for enhancing health awareness among adolescent girls. Previous studies suggested that social-media-led knowledge diffusion improved sexual practices among adolescents through increased adaptation of condom usage and other modern methods of contraception [21, 33]. Evidence from Ghana and Tanzania underlines an increasing reliance on Facebook and WhatsApp among adolescents, asserting familiarity, feasibility, confidentiality, and comfort of sharing and exchanging knowledge with other similar age group teens being the major reasons [34–36].

Evidence from a growing body of literature suggests that exposure to social media is a promising pathway to empower communities by facilitating two-way health communication and learning through diverse reproductive and behavioral change interventions or campaigns. It also facilitates interactive health discussions in private social groups, developing and strengthening online relationships with clinicians, recording health behaviors through various health surveys, and participating in diverse digital health programs, including mHealth programs [20, 22, 23, 37–41].

In our study, although exposure to social media showed a significant association with levels of SRH knowledge, educational attainment, household wealth, and place of residence (urban vs rural) strictly exerted a greater influence on it. Findings suggest that higher education potentially enables young girls to get exposed to diverse health awareness programs and internet sources, remain up to date with health recommendations and establish inner confidence and motivation, which positively affect health and knowledge-seeking behaviors. Mohan et al. (2021) recognized that even though the beneficiaries (women of reproductive age 15–49 years) receive health messages, sufficient education is a prerequisite to interpret and contextualize the content adequately through health improvements [32]. In rural areas, reduced access to SRH-related facilities affected by social stigma, interrupted and degraded services, and sparse local health facilities assumably result in poor SRH knowledge and awareness among adolescents [13, 42]. In parallel with other findings, lower odds of SRH knowledge in poor households could be attributed to a lack of or less education background, reduced exposure to health awareness programs, mass media sources, and restricted socio-cultural norms/gender role beliefs [43–45].

For other factors, although marital status showed no significant association with social media exposure, married girls were found to be at greater odds of having sufficient knowledge about sexual intercourse and pregnancy and contraceptive methods than unmarried ones. This finding is probably because married girls are comparatively more exposed to and supported by mainstream maternal health outreach programs that directly augment their awareness of adopting healthy sexual practices. These findings highlight a particular area of concern and reinforce the need to intensify efforts on more inclusive age- and context-specific adolescent health programs. Additionally, marital status showed no association with HIV/AIDS knowledge. This finding could be due to a lack of/weak HIV/AIDS-related advocacy programs, which receive minimal attention in maternal healthcare policies, a lack of awareness campaigns and social stigma, resulting in having insufficient knowledge about HIV/AIDS among adolescent girls [46].

Where restrictive social norms prevail, social media disengagement prevents girls from seeking urgent SRH information and countering misinformation/misconceptions, which can negatively affect their health-seeking behaviors and practices. When factors like logistic challenges, sub-optimal provision of services at the point of care, and social taboo inhibit adolescents from adequately seeking health information, social media provides a unique opportunity for the adolescent girls to realize their opportunities while maintaining strict confidentiality. Recent evidence from India recognizes Facebook as a rapid, cost-effective, and powerful tool in achieving socio-behavioral change, thus empowering marginalized adolescents with effective health communication and enhanced knowledge [22].

Since deep entrenched restrictive social norms restrict girls’ social mobility, digital technologies equipped with social media offer an effective channel enabling social participation, improved interaction/communication among their own social networks [30]. This would not only improve SRH knowledge, but also has a potential in improving SRH behaviours and access to related services. While we present a strong positive association with social media exposure and improved SRH knowledge, we also noticed that the knowledge gains are unevenly distributed and significantly skewed among the wealthier and educated groups. Indeed, we are cognizant of the reality that efficient social media use would not be achievable without strategic investments in education and uplifting/revamping related social and structural integrity. Our results suggests that amplification of digital technologies and its diffusion should be supplemented with improved access to quality education which empower adolescent girls both socially and economically. In addition, given the persistent rural–urban digital divide in India, establishment of digital acceptability, affordability, and accessibility to uninterrupted quality use of digital technologies requires adequate prioritisation of both social (for example, dominant gendered power dynamics and cultural context affecting digital engagement behaviour) and structural (for example, technological context and spatial marginalisation) elements. These will be exceedingly important to support the accommodation of new age technology and lowering the struggle and competition for digital inclusion.

The present study has several strengths. It provides a comprehensive understanding of the multiple socio-demographic factors linked with exposure to social media and the extent to which social media connectivity is associated with the enhancement of knowledge across three selected dimensions of SRH among adolescent girls. To the best of our knowledge, this study is the first of its kind to provide this sort of evidence that has significant potential for policymaking. Our research findings add substantial value to the current knowledge gap in the existing literature to improve and promote health-seeking behaviors by interventions of a more-focused tailored approach to acquiring adequate SRH knowledge among female adolescents. However, some limitations need to be considered in interpreting the findings of this study. First, the study was confined to two states of India and cannot be generalized to the whole country. Second, the cross-sectional nature of the study design constrains us to determine any causal relationship between social media exposure and health knowledge. Future research could use a longitudinal study design to understand this association better. Third, the data collected in the survey was self-reported and retrospective, and thus it might suffer from non-systematic reporting bias and systematic recall bias. Finally, we included those explanatory variables that were available in the dataset and, therefore, unable to include other potential socio-cultural and behavioral covariates in the multivariable models that may have a significant influence on SRH knowledge.

## Conclusion

Findings from this study indicate that exposure to social media is significantly associated with SRH knowledge among adolescent girls. Our study also found that girls who reside in urban areas, have higher levels of education and are from wealthier families are more likely to be informed about SRH knowledge than their counterparts, thus, majorly excluding adolescents from marginalized communities. While our findings present a strong association between social media and SRH knowledge, we recognize the inherent individual, social, and systemic challenges that differentially impact and deprive the most disadvantaged with fragmented or no health benefits. Thus, the policy implications of this study are two-fold. First, it will assist policymakers in future decision-making on strategic digital health investments. Second, it informs future program designing to consider multifaceted individual-level socio-demographic determinants for uniform and effective social media/digital media led SRH knowledge dissemination. To maximise benefits, ongoing and future digital media aided SRH programs should precisely identify and be mindful of contextual risk factors like socially embedded predominant patriarchal norms, interpersonal violence, which largely confine phone usage and limit individual phone ownership among married/unmarried adolescent girls [47].

## Data Availability

The data analyzed in this study was requested from the Harvard Dataverse repository through application of an official data request form. The UDAYA questionnaire is freely and publicly available from this link: https://dataverse.harvard.edu/dataset.xhtml?persistentId=doi:10.7910/DVN/ZJPKW5.

## Abbreviations

CI: Confidence interval
mHealth: Mobile health
OR: Odds ratio
SRH: Sexual and reproductive health
SC: Scheduled caste
*ST*: Scheduled tribe
OBC: Other backward classes
HIV/AIDS: Human immunodeficiency virus/acquired immune deficiency syndrome
UDAYA: Understanding the lives of adolescents and young adults
